# Factor Structure of a Multidimensional Gender Identity Scale in a Sample of Chinese Elementary School Children

**DOI:** 10.1100/2012/595813

**Published:** 2012-06-04

**Authors:** Lu Yu, Dong Xie, Daniel T. L. Shek

**Affiliations:** ^1^Department of Applied Social Sciences, The Hong Kong Polytechnic University, Hong Kong; ^2^Department of Psychology and Counseling, University of Central Arkansas, Conway, AR 72035, USA; ^3^Public Policy Research Institute, The Hong Kong Polytechnic University, Hong Kong; ^4^Department of Social Work, East China Normal University, Shanghai 200062, China; ^5^Kiang Wu Nursing College of Macau, Macau; ^6^Division of Adolescent Medicine, Department of Pediatrics, Kentucky Children's Hospital, University of Kentucky College of Medicine, Lexington, KY 40506, USA

## Abstract

This study examined the factor structure of a scale based on the four-dimensional gender identity model (Egan and Perry, 2001) in 726 Chinese elementary school students. Exploratory factor analyses suggested a three-factor model, two of which corresponded to “Felt Pressure” and “Intergroup Bias” in the original model. The third factor “Gender Compatibility” appeared to be a combination of “Gender Typicality” and “Gender Contentment” in the original model. Follow-up confirmatory factor analysis (CFA) indicated that, relative to the initial four-factor structure, the three-factor model fits the current Chinese sample better. These results are discussed in light of cross-cultural similarities and differences in development of gender identity.

## 1. Introduction

Gender identity is an integrated element of self-concept that has important implications not only on an individual's gender development but also his or her psychological adjustment [1–3]. Traditionally, researchers defined gender identity as one's identification and acceptance of him or herself [4, 5]. However, from a more contemporary perspective, gender identity has been conceptualized as a multidimensional construct which contains a variety of gender-related personality traits, attitudes, and behaviors. For example, Spence argued that the underlying structure of gender identity includes not only a basic psychological sense of belongingness to one's own sex, but also other factors reflecting an integrated, high-ordered appraisal about being male or female [2]. 

Based on Spence's work [2], Egan and Perry [6] proposed a multidimensional gender identity model, in which gender identity was conceptualized to have four different aspects: (a) membership knowledge, or one's awareness of being male or female (i.e., the traditional view of gender identity); (b) gender compatibility, defined as self-perceived gender typicality (i.e., similarity to other members of the same gender category) and feelings of contentment with one's gender; (c) felt pressure for conforming to gender stereotypes; (d) intergroup bias, the belief that one's own sex is superior to the other sex. The authors further assumed that these dimensions are more or less independent of each other and affect children's psychological adjustment. Egan and Perry developed a self-reported questionnaire to measure gender compatibility, felt pressure to conform to gender stereotypes, and intergroup bias [6]. The first dimension, membership knowledge, was not included in the measure because it had been well studied. Through exploratory factor analyses (EFA), the gender compatibility scale was broken into two aspects: gender typicality and gender contentment. Based on these results, they proposed a four-factor model of gender identity with the other two factors entitled felt pressure of gender conformity and intergroup bias.

Egan and Perry's model [6] and the psychometric properties of the measure they developed were subsequently supported by a series of studies [7–9]. For example, in a two-year longitudinal study, Yunger and colleagues [9] found that intercorrelations among the four dimensions were generally independent of each other and all the four scales had satisfying scale score reliability (Cronbach's alpha ranging from 0.70 to 0.85) and test-retest reliability (ranging from 0.40 to 0.53 with one-year interval). They also found that low gender typicality, low gender contentment, and high felt pressure measured in the first year predicted worse psychological adjustment in the second year. Moreover, a combination of high felt pressure and low gender typicality further leads to a deterioration of participants' psychological well-being [9].

In spite of the above support for the model and its measure, several important issues have remained unresolved. First, the four-factor structure of Egan and Perry's measure [6] has not been subjected to extensive work based on factor analysis, either by Eagan and Perry or by other researchers. Egan and Perry only performed EFA on gender compatibility and felt pressure but not on the intergroup bias scale [6]. Moreover, no confirmatory factor analysis (CFA) has been used to confirm the established factor structure of the measure.

Further, whether Egan and Perry's model can be applied to cultures other than America remains unclear. Corby, Hodges, and Perry's study [10] suggested that the four-factor gender identity model may lack generalizability to other cultures. They further argued that Egan and Perry's model may need some amendments, and additional dimensions may need to be considered for gender identity development in other cultures. The contextual effects on social identity have long been emphasized in that the social context not only prescribes the stereotypes concerning specific social groups but also affects the way people see themselves and others [11, 12]. The embodiment of contextual effects on gender identity involves the culture-specific gender stereotypes, social status of the two sexes, and various patterns of gender socialization in different societies [13, 14].

In spite of the emphasis on cultural influences on gender development and identity formation [15–17], few empirical studies have attempted to examine the multidimensional gender identity model cross-culturally. This type of research is important for expanding current knowledge about gender socialization and promoting children's positive psychological development in more cultural-effective ways.

China encompasses roughly one-fifth of the world population, and more than 200 million children under 12 years old in the PRC constitute 18% of all the world's children in this age group [18]. Besides, it has a very distinct cultural context from Western cultures represented by the United States. Researchers have reported cross-cultural differences between Chinese and Western populations in terms of gender stereotypes and gender role development [16, 19]. For example, Yu and Xie reported that the communal traits, almost exclusively defined as feminine characteristics in Western cultures, were incorporated into both feminine and masculine traits in Chinese culture [20]. However, no empirical study has yet examined the multiple components of gender identity in the Chinese culture. Most of the existing studies on Chinese children's gender identity-related issues [21–23] only focused on describing the developmental trends of gender stereotyping and basic processes of gender identity formation such as gender label, gender constancy, and gender schematics. Chinese children's gender identity has yet to be studied from a multidimensional perspective. There are also researchers pointing out the lack of assessment tools on the psychosocial functioning of Chinese children and adolescents [24, 25].

The present study is the first to apply Egan and Perry's gender identity model and their measure to the Chinese culture. The purpose of this study was to examine the factor structure of Egan and Perry's gender identity measurement on a sample of Chinese elementary school students. We hope this study will provide not only a better understanding of the multidimensional structure of gender identity in Chinese children, but also a useful measure for empirical studies on Chinese children's gender identity development.

## 2. Methods

### 2.1. Participants and Procedures

Participants were 726 third to sixth graders randomly sampled from three elementary schools in a city of eastern Mainland China, with a population approximating 4.6 million. Among the participants, there were 395 boys (M(age) = 10.46, SD = 1.31) and 331 girls (M(age) = 10.57, SD = 1.28). In order to conduct EFA and CFA on different samples, all participants were randomly divided into two groups: Group 1 contained 366 participants, with 191 boys and 175 girls; Group 2 included 360 participants, with 204 boys and 156 girls. In classroom settings, the children were asked to complete a survey about how they think about themselves, and then the translated Egan and Perry's measure [6] of multiple gender identity components was administered over a 45-minute period. For the third and fourth graders, each item was read aloud by the researcher to ensure correct understanding of its meaning.

### 2.2. Instruments

Egan and Perry [6] developed a 34-item self-reported gender identity measure that assesses gender typicality, gender contentment, felt pressure for gender conformity, and intergroup bias. For each item, children first decided which of the two kinds of children described in the item they were like more and then indicated whether this choice was *very true or sort of true* for them. The reported Cronbach's *α* coefficients for the four scales on samples of children in the United States were all around 0.80 [7].

Egan and Perry's measure [6] was translated into Chinese and backtranslated into English to ensure linguistic and conceptual equivalence [26]. The original English versions of these instruments were first translated into Chinese. Then, the translated Chinese versions were backtranslated into English by a second bilingual person. The two English versions were compared, and the two translators discussed the discrepancies. In order to determine both linguistic and conceptual equivalence between the two cultures, we then consulted with both native speakers of English and Chinese mandarin who have proficient knowledge of their respective languages and cultures and made necessary revisions.

## 3. Results

Data analyses involved four steps. First, psychometric properties of the four-scale gender identity measure were examined on Group 1 participants. In the second step, exploratory factor analyses (EFA) were conducted on Group 1 participants to explore the factor structure of the gender identity structure on Chinese participants. The third step involved structural equation modeling to cross-validate the new factor structure and compare the model fit of the new factor structure and the original four-factor structure [6] using Group 2 participants. Finally, the psychometric properties of the subscales based on the new factor structure of gender identity measure were examined on Group 2 participants.

### 3.1. Psychometric Properties of the Original Four-Factor Gender Identity Measure

Table 1 presents the internal consistency coefficients of the four subscales in the gender identity measure based on Group 1 participants. Overall, the Cronbach's *α* values in this Chinese sample were not as high as those reported in previous studies based on American samples. Specifically, gender contentment (*α* = 0.50) and gender typicality (*α* = 0.61) scales did not seem to be cohesive gender identity dimensions in the current sample. Although the alpha reliabilities of felt pressure (*α* = 0.77) and intergroup bias (*α* = 0.71) scales were acceptable, they were still lower than those reported in previous studies [6, 9].

Table 1 also presents correlation coefficients among the four subscales based on Group 1 participants. There was a significant correlation between gender typicality and gender contentment (*r* = 0.45, *P* < 0.001). The correlations between gender contentment and felt pressure as well as between felt pressure and intergroup bias were also statistically significant, with a low to medium effect size.

### 3.2. Exploratory Factor Analysis (EFA)

The 34 items of Egan and Perry measure of gender identity [6] were subjected to EFA to explore the factor structure of gender identity for Chinese participants. Based on the 366 participants in Group 1, maximum-likelihood (ML) factor analysis was conducted. As suggested by Fabrigar et al. [27], ML is the best method of extraction in EFA for relatively normally distributed data because ML permits the computation of a series of goodness-of-fit indexes of the model and allows for statistical significance testing of factor loadings as well as correlations among factors. The results showed 10 factors with eigenvalues exceeding 1, explaining 55.28% of the total variance. However, an inspection of the scree plot showed a break after the third factor. Parallel analysis (Table 2) showed that the first four factors had eigenvalues greater than the corresponding criterion values for a randomly generated data matrix of the same size (34 items × 366 participants). While parallel analysis suggested a four-factor solution, the actual eigenvalue of the fourth factor (1.47) was only slightly greater than its corresponding randomly generated eigenvalue (1.44) in contrast to the differences on the third factor (2.32 versus 1.48). Therefore, a three-factor solution might be more appropriate.

Table 3 presents the pattern/structure matrix of the three-factor solution. The three-factor solution explained a total of 29.38% of the variance, with Factor 1 contributing 11.72%, Factor 2 contributing 8.90%, and Factor 3 contributing 8.76%. The Varimax rotation and Kaiser Normalization were used to aid in the interpretation of these three factors. This rotated three-factor solution yielded moderate to strong factor loadings (>0.30), and most of them loaded on only one factor. This suggested the presence of a simple structure [28]. The interpretation of the three factors was consistent with Egan and Perry's original multidimensional gender identity model [6]. Specifically, the 14 items loading on the first factor denoted pressure of conforming to gender stereotypes; the eight items on the second factor reflected intergroup bias; the 13 items loading on the third factor focused on gender compatibility. These three factors were the major gender identity components Egan and Perry originally proposed [6]; however, their subsequent factor analysis suggested that the dimension of gender compatibility can be better conceptualized as two dimensions (i.e., gender typicality and gender contentment). Based on this result, Eagan and Perry modified their model as a four-factor structure of gender identity. However, the EFA in the present study suggested a three-factor structure for the Chinese participants. CFA was used, to subsequently examine the model fit of this three-factor structure and Egan and Perry's four-factor structure [6] in the Chinese culture.

### 3.3. Confirmatory Factor Analysis (CFA) of the Four-Factor and the Three-Factor Structure of Egan and Perry's Gender Identity Measure

**Figure 1:**
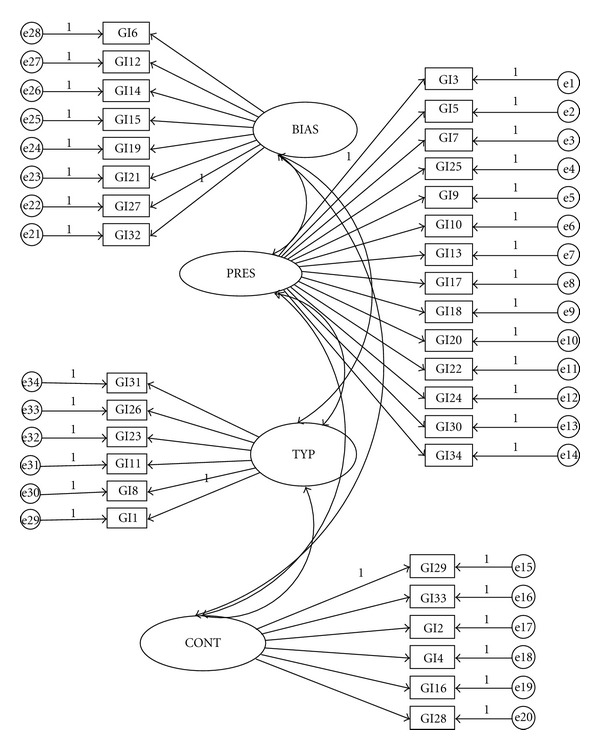
Path diagram for the correlated four-factor model. Note: BIAS: intergroup bias; PRES: felt pressure; TYP: gender typicality; CONT: gender contentment. GI: gender identity measure item.

**Figure 2:**
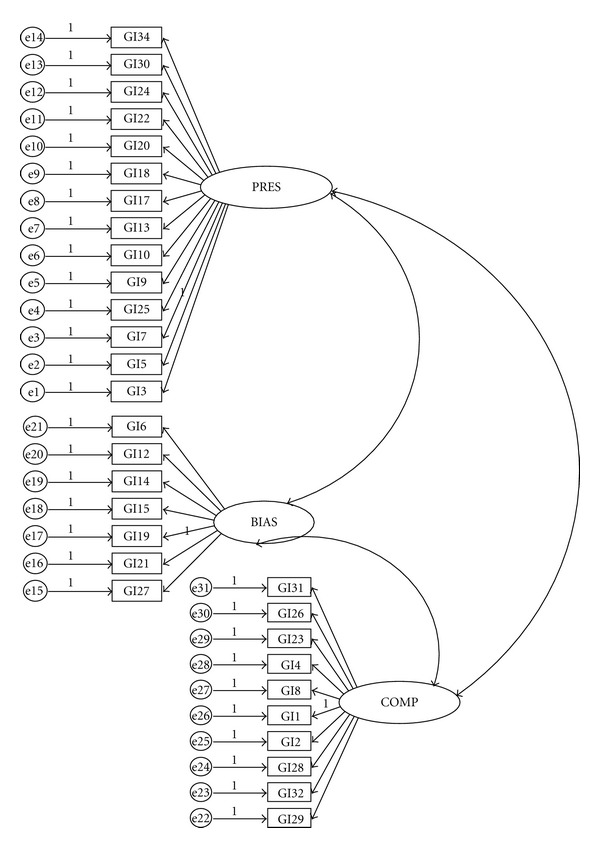
Path diagram for the correlated three-factor model. Note: BIAS: intergroup bias; PRES: felt pressure; COMP: gender compatibility. GI: gender identity measure item.

Using AMOS 4.0 in SPSS version 15.0, two CFAs with maximum likelihood (ML) estimation were performed on items of gender identity measure on 360 participants in Group 2 to evaluate the model fit of the three- and four-factor models. The result of EFA showed that item 33 loaded on two factors, and items 11 and 16 had low loadings; therefore, these items were excluded from the original 34 items when testing the three-factor model. In both models (Figures 1 and 2), the factors were hypothesized to covary with one another in that they all reflect one's integrated judgment about being one sex. Circles represent latent variables, and rectangles represent measured variables. Absence of a line connecting variables implies no hypothesized direct effect.

To evaluate the overall fit of the models, several fit indices were employed. These included chi-square (*χ* ^2^), goodness-of-fit index (GFI), comparative fit index (CFI), nonnormed fit index (NNFI), Akaike's information criterion (AIC), consistent Akaike's information criterion (CAIC), expected cross-validation index (ECVI), root mean square residual (RMR), and root mean square error of approximation (RMSEA) [29, 30]. The main model fit indexes are presented in Table 4.

Several observations can be highlighted from the results. First, the two absolute fit indices showed a better fit to the data for the three-factor model (*χ* ^2^ = 878.90 and GFI = 0.89) than the four-factor model (*χ* ^2^ = 1057.39 and GFI = 0.85). Second, values of incremental fit indices (CFI and NNFI) for the three-factor model were higher than those for the four-factor model, indicating that the three-factor model had a higher relative improvement compared to the baseline model. Third, in terms of measures based on residual correlations, the three-factor model had lower values on both RMSEA (0.05) and RMR (0.07) than did the four-factor model (RMSEA = 0.06; RMR = 0.08), which suggests that the three-factor model had lower model fit residuals. Finally, the three-factor model is more parsimonious than the four-factor model with smaller values of AIC, CAIC, and ECVI. Thus, taken all these results together, the three-factor model appeared to fit the data better for Chinese participants than the previous four-factor model [29, 31]. The improvement in fit between the three-factor model and four-factor model was statistically significant (Δ*χ* ^2^ = 178.1, Δ*df* = 90, *P* < 0.001).

### 3.4. Psychometric Properties of the New Three-Scale Gender Identity Measure

Based on the three-factor model, three subscales were generated for the Chinese sample, namely, the gender compatibility scale, felt pressure scale, and intergroup bias scale.  Table 5 presents Cronbach's *α* coefficients and the intercorrelation coefficients of the three subscales. Compared to the original four subscales based on the four-factor structure, the three subscales overall have higher internal consistency and were more independent of each other. This further supported the reliability and validity of the three-factor model for the Chinese culture.

## 4. Discussion

Egan and Perry [6] originally proposed gender compatibility as a single component of gender identity, which was defined as self-perceived gender typicality (i.e., similarity to other members of the same gender) and feelings of contentment with one's gender. However, their subsequent factor analysis suggested that this component could be better conceptualized as two separate components: gender typicality and gender contentment. Together with the felt pressure of conforming to gender stereotypes and the intergroup bias (i.e., the belief that one's own sex is superior to the other sex), they proposed a four-factor model and a measure assessing these four gender identity components. However, after applying the model and the measure to the Chinese samples, the present study found that a three-factor structure of gender identity may fit the Chinese culture better than the original four-factor structure that was based on the American samples. Both supporting and opposing evidence were found for cross-cultural consistency in the related factor structure. While the structures of felt pressure and intergroup bias may share some similarities cross-culturally, gender typicality and gender contentment are less differentiated from each other under the Chinese culture than under the Western cultures.

The present study found that gender contentment (i.e., feeling satisfied about one's sex) may not be a significant aspect of gender identity for Chinese individuals, relative to that of individuals in Western cultures. This is consistent with some characteristics of the Chinese culture. Positive self-regard or self-satisfaction is not an important component of the self structure [32–34]. In the Chinese culture, being content with oneself is often considered undesirable and has negative implications [35, 36]. This cultural norm of devaluing self-contentment is evidenced in many historical and prominent proverbs found within Chinese societies. For example, “*qian shou yi, man zhao sun*,” translated as “modesty is the cause of gain, and complacency is the root of loss,” and “*yi ri san xing*,” translated as “inspect oneself in three regards everyday.” The existence of this cultural norm has also been supported by empirical studies. For example, Eid and Diener [37] found that pride and contentment were valued positively by people from individualistic cultures such as Australia and the United States but were regarded as negative and undesirable by participants from Mainland China. Guided by this cultural norm, Chinese individuals tend to focus more on their current inadequacy in effort for self-improvement, as opposed to developing a sense of satisfaction with oneself. The finding of the present study that gender contentment is not an independent facet of gender identity may highlight this cultural-specific value.

It is also possible that, for Chinese children, the composition associated with gender contentment might be conceptually related to the composition associated with gender typicality. In contrast to individualistic societies, people's evaluations and feelings about themselves in collectivism cultures are largely based on their interpersonal relationships which assume priority over the individual attributes [38–40]. In a similar vein, the extent to which an individual feels satisfied with his or her sex would be closely related to one's connectedness with other member of the same sex. Meanwhile, being congruent with social norms is highly emphasized in most collectivistic cultures [41]. Individuals grown up under these cultures are socialized to identify with and conform to social expectations and norms of the groups they belong to and adjust themselves to better fit in these groups [42]. Therefore, high self-perceived gender typicality would help to elevate one's acceptance gained from the group, which, may contribute to enhancing one's gender contentment. Because of the possible connection between these two aspects, gender typicality and gender contentment did not stand out as two independent factors of gender identity but rather stayed as one unified factor for the Chinese participants.

Another interesting finding is about item 32, which read as *“some boys (girls) do not think that boys (girls) are more friendly than girls (boys), but other boys (girls) do think that boys (girls) are more friendly than girls (boys).” *This item was originally in the intergroup bias scale in Egan and Perry's measure; however, it now had a higher loading on the new gender compatibility factor (0.41) than on the intergroup bias factor (0.29) for the Chinese children. This suggested that for Chinese children, the belief that boys (girls) are better than girls (boys) is more likely to reflect a sense of being a typical member of his or her gender group, or being compatible with his or her ingroup, rather than a discriminatory attitude toward their outgroup counterparts. This may also highlight the importance of maintaining interpersonal harmony in Chinese children's perceived overall gender compatibility. For example, when a boy considers other boys as more friendly than girls, he may feel that he relates well to his same sex peers and perceive himself as being compatible with other boys. This perception may not necessarily lead to the boy's negative attitude toward girls.

When applied to Chinese children, the three scales (gender compatibility, felt pressure, and intergroup bias) all demonstrated moderate internal consistency. Especially for the combined gender compatibility scale, its Cronbach's alpha coefficient was greater than either the gender typicality scale or gender contentment scale in the original four-factor structure. This supported the reliability of this combined scale. The relationships among the three scales were from nonsignificant to moderate, which suggest adequate discriminant validity for this three-scale measure. In other words, as specified by the gender identity model [6], these scales measure related but still separate aspects of gender identity for Chinese participants.

In summary, the present study overall supported the application of Egan and Perry's multidimensional gender identity model [6] under the Chinese culture. Factor analysis on Egan and Perry's measure suggested a three-factor structure on the current Chinese sample, which is similar but different from the proposed four-factor gender identity model for American children. Gender contentment and gender typicality might be better conceptualized as a combined dimension for Chinese children, rather than as two independent facets of gender identity for American children. The present study also indicated that the three-scale gender identity measure has satisfactory psychometric properties for Chinese children.

However, the present study has several limitations. First, only self-reported data were collected in this study, in which participant's response bias may compromise the results. Data from more comprehensive sources such as parents, teachers, and peers should be collected in future research. Secondly, the present study did not assess Chinese children's gender-related behavior or personality traits, which otherwise would provide convergent and divergent validity for the three-scale gender identity measure. Future research may need to examine the relationships between the three gender identity components and sex-typing indices, such as whether or not the exhibited gender typical behavior is associated with one's perceived gender typicality. Third, the results were interpreted from a cross-cultural perspective but did not directly compare cultural differences regarding the construct of the multidimensional gender identity. Subsequent research may incorporate participants from other cultures and focus on the direct comparison of the factor structure of gender identity. Finally, it should be noted that the participated children in this study were recruited from a big Chinese city. Given that the discrepancy exists in social environment and subculture between urban and rural areas in China, findings of the current study should be interpreted with cautious and may not be readily generalized to children living in rural regions. Further understanding of the construct of gender identity will require inclusion of a larger sample of children with diverse social backgrounds.

In spite of the above limitations, the present study provided evidence for the cross-cultural applicability of the multidimensional model of gender identity to Chinese population. The revised three-scale measure of gender identity appeared to be a promising instrument for future research concerning Chinese children's gender identity. This is a constructive response to the criticism of Shek [24, 25] that there is a severe lack of psychosocial measures in the Chinese culture. Researchers and clinicians can use this instrument to investigate the developmental process of children's gender identity formation from a multidimensional perspective. With this instrument, they can also examine the determinants and correlates of different gender identity components and to further explore the effects of gender identity on other aspects of Chinese children's psychological development. Practically speaking, the developed scale can also help to evaluate positive youth development programs with focus on positive identity and prosocial norms, where gender identity will be covered [43, 44].

## Figures and Tables

**Table 1:** Internal consistency reliability of the four-scale gender identity measure for the current Chinese sample and zero-order correlations among scales.

Scales	Alpha		Correlations		
	Chinese sample (*n* = 366)	American sample^a^ (*n* = 235)	TYP	CONT	PRES
TYP	0.61	0.78	1		
CONT	0.50	0.79	0.45**	1	
PRES	0.77	0.92	0.03	0.16**	1
BIAS	0.71	0.73	0.05	−0.03	0.28**

Note: TYP: gender typicality scale; CONT: gender contentment scale; PRES: felt pressure scale; BIAS: intergroup bias scale *P < .05. **P < .01.

^a^Alpha coefficients from Egan & Perry's study [6] was listed for the purpose of comparison.

**Table 2:** Comparison of eigenvalues from the actual factor analysis and random eigenvalues from parallel analysis.

Factor number	Actual eigenvalues	Random eigenvalues
1	4.61	1.62
2	3.06	1.54
3	2.32	1.48
4	1.47	1.44
5	1.37	1.38

Note: Parallel analysis was based on Watkins [45]. Number of variables: 34; number of subjects: 366. Only the first five eigenvalues were presented.

**Table 3:** Structure matrix for maximum-likelihood factor analysis with varimax rotation of three-factor solution of Egan and Perry's [6] gender identity measure.

Item	Content	Factor loadings		
		Factor 1	Factor 2	Factor 3
7	Think the boys (girls) they know would be upset if they wanted to play with girls' (boys') toys	**0.64**	−0.08	−0.04
18	Do not think other boys (girls) would be upset if they want to learn how to knit or sew (fish or hunt)	**−0.63**	0.12	−0.10
30	Think their parents would be upset if they wanted to play with girls' (boys) toys	**0.61**	−0.09	0.10
34	Think the boys (girls) they know would mind if they wanted to take ballet lessons (fix cars or bicycles)	**0.56**	−0.23	0.03
13	Do not think other boys (girls) would be upset if they wanted to learn an activity that only girls (boys) usually do	**−0.52**	−0.02	0.07
5	Get really mad if someone says they're acting like a girl (boy)	**0.50**	−0.17	−0.22
9	Think their parents would be upset if they wanted to learn an activity that only girls (boys) usually do	**0.50**	0.00	0.01
17	Do not think other boys (girls) would be upset if they wanted to play or talk with girls (boys)	**−0.49**	0.22	−0.16
24	Try hard to do all the things boys (girls) are supposed to do	**0.44**	0.09	−0.24
22	Do not really care if they are like other boys (girls) they know	**−0.44**	0.06	0.13
10	Do not think their parents would be upset if they wanted to learn how to knit or sew (fish or hunt)	**−0.39**	0.03	−0.22
20	Do not think their parents would mind if they wanted to take ballet lessons (fix cars or bicycles)	**−0.37**	0.18	−0.05
3	Do not think it is important to act just like other boys (girls)	**−0.35**	−0.09	0.13
25	Do not think their parents would be upset if they wanted to play or talk with girls (boys)	**−0.32**	0.20	−0.25
21	Think that boys (girls) are more honest than girls (boys)	0.09	**−0.69**	0.03
19	Think that girls (boys) are more annoying than boys (girls)	0.09	**−0.68**	−0.02
27	Do not think that boys (girls) are more creative than girls (boys)	−0.19	**0.64**	0.07
6	Do not think that boys (girls) are more truthful than girls (boys)	0.00	**0.64**	0.07
15	Think that girls (boys) are not as smart as boys (girls)	0.18	**−0.59**	0.15
14	Do not think that girls (boys) are more lazy than boys (girls)	−0.01	**0.51**	0.02
12	Think that girls (boys) are more boring than boys (girls)	0.19	**−0.35**	−0.02
33	Do not mind that some things are only for girls (boys)	0.06	**0.31**	**−0.30**
31	Do not feel that their personality is similar to other boys' (girls') personalities	−0.04	−0.03	**0.66**
26	Feel that the things they like to do in their spare time are different from what most boys (girls) like to do in their spare time	−0.09	−0.02	**0.66**
23	Do not feel they are just like all the other boys (girls) their age	0.01	−0.12	**0.57**
8	Do not feel they fit in with other boys (girls)	0.22	0.05	**0.46**
4	Like being a boy (girl)	0.27	0.03	**−0.45**
29	Never think it might be more fun to be a girl (boy)	0.29	−0.03	**−0.44**
28	Wish it would be okay for them to do some of the things that usually only girls (boys) do	−0.26	0.07	**0.43**
32	Do not think that boys (girls) are more friendly than girls (boys)	0.20	.29	**0.41**
2	Feel annoyed that they're supposed to do some things just because they are a boy (girl)	0.11	−0.13	**0.35**
1	Feel that the kinds of things they're good at are similar to what most boys (girls) are good at	0.09	−0.07	**− 0.34**
11	Think they are a good example of being a boy (girl)	−0.04	−0.12	−0.29
16	Never feel cheated that there are some things they're not supposed to do just because they're a boy (girl)	−0.12	0.11	−0.23

Note: The items of Egan and Perry's gender identity measure are descriptions of two types of “kids” written in the format developed by S. Harter and S. Harter [46]. For example, the full content of item 19 is “Some boys (girls) think the boys (girls) they know would be upset if they wanted to play with girls' (boys') toys, but, other boys (girls) do not think the boys (girls) they know would be upset if they wanted to play with girls' (boys') toys.” Due to the limited space, only the first half parts of the items were presented in the above table. Item 19, as an example, was presented as “Think the boys (girls) they know would be upset if they wanted to play with girls' (boys') toys.” Interested readers may refer to Egan and Perry's literature [6] for the English version of the questionnaire. The Chinese version of the measure is available from the correspondence author upon request.

**Table 4:** Fit indices for the two models.

Model	*χ* ^2^	*df*	RMR	AIC	CAIC	CFI	RMSEA
Four-factor model	1057.39	521	0.08	1205.39	1567.17	0.69	0.06
Three-factor model	878.90	431	0.07	1008.90	1326.68	0.78	0.05

**Table 5:** Internal consistency reliability and correlations among the three-factor gender identity measure based on the Chinese sample (*N* = 360).

	Number of items	Cronbach's alpha	COMP	PRES	BIAS
COMP	10	0.67	1	—	
PRES	14	0.77	0.12*	1	
BIAS	7	0.73	−0.02	0.22**	1

*Note*: *N* = 360; COMP: gender compatibility scale; PRES: felt pressure scale; BIAS: intergroup bias scale *P < 0.05, **P < 0.01.
